# Smart Spider Monkey Optimization (SSMO) for Energy-Based Cluster-Head Selection Adapted for Biomedical Engineering Applications

**DOI:** 10.1155/2022/2538115

**Published:** 2022-01-30

**Authors:** P. Ajay, B. Nagaraj, J. Jaya

**Affiliations:** ^1^Faculty of Information and Communication Engineering, Anna University, Chennai, India; ^2^Department of ECE, Rathinam Technical Campus, Coimbatore, India; ^3^Department of ECE, Hindusthan College of Engineering and Technology, Coimbatore, India

## Abstract

Using energy efficiency to increase the life and sustainability of wireless sensor networks (WSNs) for biomedical applications is still a challenge. Clustering has boosted energy productivity by allowing cluster heads to be categorized, but its implementation is still a challenge. Existing cluster head selection criteria start with determining acceptable cluster head locations. The cluster heads are picked from the nodes that are most closely connected with these places. This location-based paradigm incorporates challenges such as faster processing, less precise selection, and redundant node selection. The development of the sampling-based smart spider monkey optimization (SSMO) approach is addressed in this paper. If the sample population's nodes are varied, network nodes are picked from among them. The problems with distributed nodes and cluster heads are no longer a concern. This article shows how to use an SSMO and smart CH selection to increase the lifetime and stability of WSNs. The goal of this study is to look at how cluster heads are chosen using standard SMO and sampling-based SMO for biomed applications. Low-energy adaptive clustering hierarchy centralized (LEACH-C), particle swarm optimization clustering protocol (PSO-C), and SSMO improved routing protocol measurements are compared to those obtained in homogeneous and heterogeneous settings using equivalent methodologies. In these implementations, SSMO boosts network longevity and stability periods by an estimated 12.22%, 6.92%, 32.652%, and 1.22%.

## 1. Introduction

WSNs were used in a variety of applications including smart homes, disaster monitoring, air purifiers, and so on, due to their increased productive output, convenience of the use, and reduced price. Sensor nodes are occasionally placed in dangerous areas, making it difficult to replace batteries or repair malfunctioning nodes. Additionally, improving a node's battery performance incurs additional costs. As a result, research has concentrated on extending the life and stability of networks through the use of a variety of different communication protocols [1]. Through the use of clustering, the low-energy adaptive clustering hierarchy (LEACH) protocol maximizes energy efficiency. The distance between nodes and base stations (BSs) has an effect on the amount of energy consumed during data transfer. Clustering is based on the principle of reducing the difference between nodes that are not cluster heads (CHs), which gather information from neighbouring nodes for forwarding. As a result, selecting the appropriate CH enables more smart energy consumption. In LEACH, nodes are selected as CHs at random, regardless of their state or character traits, such as residual energy, expected energy usage, or number of nearest neighbours. When selecting CHs, it is necessary to consider the centrally controlled use of understanding on all nodes at the BS. However, simultaneous acquisition of all of this data at the BS via transfer is challenging. To obtain available power information from LEACH-C nodes, node information is synced using time-division data transmission techniques. LEACH-C not only increases data availability but also empowers the BS to have more computational power than the nodes. As a result, this type of centralized operation could be used to increase the effectiveness of clustering. Swarm intelligence-based clustering is an amazingly precise method that is widely adopted in optimal control protocols. This strategy has been implemented in a number of different protocols, such as particle swarm optimization (PSO), bee colony optimization, as well as ant colony optimization. In comparison to certain other swarm intellect optimization algorithms, the recently developed spider monkey optimization (SMO) algorithm is inspired by the behavioral patterns of spider monkeys in search of food in order to smartly and precisely decide on feasible solutions [2]. As a result, various studies have used SMO to select CH. In this survey, we modified the SMO automated system to further focus on improving CH selection. In the variety of operational experiments that use cluster analysis for WSNs, the nodes closest to the objective function are classified as CHs during usage. Thus, clustering is particularly concerned with locating CHs at the data point, and procedure problems can arise when optimized CH locations are considerably different from actual node locations. To begin, when the nearby nodes are determined using CH definitions, the algorithm consumes additional space, increasing energy efficiency and lowering the lifespan of the network. Second, if the optimal location of the CH node differs considerably from the actual location of the CH node, a node belonging to another cluster may be mistakenly used as the CH. Finally, a node may be selected as the cluster's CH based on its nearness to the cluster's optimal location. As a result, the CH nodes will be fewer than the cluster nodes, resulting in suboptimal performance. As a result, aggregation must be modified to take WSN attributes into account, including actual node locations. We modified SMO in this study by incorporating sampling techniques for channel access in WSNs. When sampling a population of nodes, everyone's true destinations are always extracted, avoiding the divergence between both the optimal CH location as well as the true node location previously described. As a result, the sampling process omits multiple CH selections across distinct categories, eliminating the need for distributed processing. Indeed, the modified SMO achieves optimal performance only with the finest samples (i.e., actual node destination in the world), as it differs from the conventional SMO only in that its search is sample-based. To begin, we will discuss sampling-based SMO and its application to WSNs, which include sampling-based SSMO. Additionally, SSMO is compared to form protocols to demonstrate the time-dependent effect of CH selection and node energy consumption. These findings demonstrate that when compared with the existing protocols such as LEACH-C, PSO-C, and SMOTECP, SSMO dramatically increases the average lifespan and reliability of WSNs [3].

The following individuals contributed greatly to the work.

To our knowledge, it is the first time a sampling method has been used to prolong the range and dependability of wireless sensor nodes. It suggests a sampling-based SMO and a method for selecting the most energy-smart CH (SSMO). By implementing the SSMO change, we can increase the lifetime of the network and its stability. To assess our protocol's quality, we compare it to others such as LEACH-C, PSO-C, and SMOTECP. This manuscript is divided into the following. The second section summarizes related work. The article examines SMO based on sampling, and the Section 4 mentions the proposed SSMO protocol.  Section 5 summarizes the study data, equates SSMO to comparable protocols, and concludes with a brief. Finally, we present our findings in Section 6.

## 2. Related Work

LEACH optimizes the efficiency of wireless sensor networks by utilizing clustered hierarchical networks. Clustering involves assigning a data collection destination to each cluster (i.e., CH). Likewise, a predictive method is used to identify the CH, but node-specific information, such as power consumption, is omitted [4]. To make use of the data stored on other nodes, this must be transferred, but doing so over wireless networks is challenging. LEACH-C overcomes this limitation through the use of time-division data transmission. The BS informs each CH of the outcome of the CH selection and communicates transmission. Additionally, the CHs converse with adjacent nodes and convey schedules in order to minimize latency. LEACH-C prioritizes CHs at nonsensor nodes that have low computing costs. Due to the fact that the BS and other elements perform the computations required for CH selection, it is possible to utilize a large amount of computer power. LEACH maximizes the energy efficiency of WSNs by utilizing clustered hierarchical networks. Clustering assigns a data collection location to each cluster (i.e., CH). Furthermore, a predicting future model is used to calculate the CH, but node-specific information such as residual energy is omitted. To access the data stored on other nodes, it should be transferred, but transmitting and receiving those very data over wireless connections is challenging. LEACH-C overcomes this limitation through the use of time-division multiple access [5]. The BS informs each CH of the end result of the CH selection process and integrates transmission. Additionally, the CHs communicate with their nearest neighbours and transmit schedules to minimize latency. LEACH-C prioritizes CHs at nodes with low computing costs other than sensor nodes. Because the BS and other components preform computations for CH selection, computer and Internet resources can be utilized.

We adjusted the quantity of data received and the node to compensate for each CH's coverage area, suggesting that the majority of sensor nodes are evenly distributed throughout the WSN. Thus, if coverage across CHs is comparable, they access data from a comparable number of nodes. As a result, unless a node openly selects a CH, the volume of data obtained can be adjusted. By defining the coverage area and devoting energy centers to the CHs, PSO-EC establishes the energy distribution network [6]. By choosing the node with the highest energy value between nearby nodes as CH, energy efficiency is increased. Because this method is related to energy dispersion, it underperforms at the start point when node energy is spread equally. SMO-C is an SMO-based protocol that, like PSO-C, optimizes this same location of the CH assigned to the nearest centroid. Two fitness values comprise the objective function: the node–CH distance and the power consumed by nodes and CHs. When data is sent to a node to the CH, the energy consumption is determined by the distance between the network's nodes. As a result, more simulations are necessary than in other protocols to obtain the fitness values, and the results do not demonstrate a substantial improvement over what would be required. Indeed, as clearly explained, SMO-C does not outperform LEACH statistically. Alternatively, by specifying this same data transmission between CHs, SMOEC has been shown to improve SMO-C. While the cable network lifetime is increased, stability is preserved due to the early deterioration of energy by certain nodes.

When PSO or SMO clustering is used, a specific area for a CH is first determined. CH is then defined as that of the node that has the strongest connection to this location. SMOTECP automatically optimizes CH selection, obviating this need for additional computation [7]. Binary SMO is used to treat CH collection as a binary problem, with the origin node receiving a label of 1 and the primary benefit receiving a label of 0. This method, on the other hand, is incapable of dealing with the number of CHs, as Boolean operations return a large number of ones (i.e., CHs), which can affect the fitness function and thus jeopardize optimization. Additionally, SMOTECP is difficult to implement in connection with a great amount of CHs.

As a result, in this study, we addressed a few critical factors affecting CH selection:

1. The objective function's energy efficiency is increased by including fitness values in addition to energy consumption
2. To optimize the set of devices covered by each CH, an objective function for both the cover areas is included in the optimization problem
3. The procedure can be used even if all nodes have the same amount of energy (initial state)
4. By selecting nodes directly, unnecessary operations are avoided
5. The amount of CHs released into the environment can be forecasted and controlled

### 2.1. Optimization Methods That Tried for the WSN Problem

Optimization is a process of increasing the dimensionality of a facility necessary for the smart operation of a system. Numerous optimization techniques are used in a variety of applications. The criteria for processing and optimization methods include maximum, minimum, and any other specified values. The optimization techniques keep track of the decision variables defined by the problem, application, or system. These objective functions are then steered in the direction specified by the aforementioned criteria. Optimization methods are required to guide the PID controller toward its optimal tuning state. The appropriate list of optimization methods for this process is as follows: (1) CTOA – class topper optimization technique, (2) GWOA – g000rey wolf optimization algorithm, (3) SRA – sequential randomized algorithm, and (4) BSOA – brainstorming optimization algorithm.

#### 2.1.1. CTOA (Class Topper Optimization Algorithm)

CTOA (class topper optimization algorithm) [8] is an optimization algorithm inspired by classroom instruction and processing. It is structured in the manner of a student attempting to earn a high grade through learning. Students in each section will compete for the position of section topper (ST), and section toppers will compete for the position of class topper (CT). The target is attained based on their individual competing and learning abilities. Generally, these toppers are chosen based on an examination-based evaluation. CTOA operates according to this methodology. CT and ST collaborate to improve the quality of knowledge shared. The topper's position attracts student's/element values in the optimization method, which finds a solution that meets the application requirement. According to the optimization problem, an objective function is defined and used to guide the iterative process by which the CTOA discovers the optimal solution through the observations it makes during each iteration.

#### 2.1.2. GWOA (Grey Wolf Optimization Algorithm)

GWOA (grey wolf optimization algorithm) is another algorithm developed through observation of nature, specifically the life of a wolf and its pack. The wolf's natural instinct is to hunt in packs. Unlike other animals, the wolf must adhere to an absolute rule when managing its pack, that is, the wolf that leads the other wolves is called alpha, and he or she has the leading authority to regulate the other wolves [9]. The beta wolf receives and executes the alpha's command. Often, the beta omega is the leader of the pack, while delta wolves are at the third level, where the delta has the option of dominating the omega. As with other optimization methods, the GWOA seeks the optimal solution in the same way that a wolf pack seeks food. The grey wolf's activities are classified into three categories, including (1) the process of locating prey (tracking process), (2) the act of rounding up the victim in order to prevent his or her escape (encircling), and (3) The act of attacking a victim for the purpose of hunting (attacking).

The encircling nature of the grey wolves

(1) $$\begin{array}{c} E^{⟶}=\left|Cf^{⟶}.P_{v}{\left(t\right)}^{⟶}-P{\left(t\right)}^{⟶}\right|,\\ P^{⟶}\left(t+1\right)=P_{v}{}^{⟶}\left(t\right)-a^{⟶}.E^{⟶}. \end{array}$$

Given that this is an iterative process, the term *t* denotes the current iteration *E*^⟶^. *P*^⟶^(*t*+1) Defines the wolf pack's encircling nature. *P*_*v*_(*t*)^⟶^ and *P*(*t*)^⟶^ are coordinate vectors that indicate the location of prey and the wolf. *Cf*^⟶^and *a*^⟶^ are the vectors of cosmarts used to approximate the wolf behavior mathematically

(2) $$\begin{array}{c} a^{⟶}=2A^{⟶}.r1^{⟶}-A^{⟶},\\ Cf^{⟶}=2.r2^{⟶}, \end{array}$$

where *A*^⟶^ varies from 2 to 0 linearly.

Because the GWOA is used in real-time optimization applications, the random quantity vectors *r*1^⟶^and *r*1^⟶^ are included in the cosmart calculation. The wolf is capable of noting and comprehending the prey's position in the area defined by each wolf hierarchy [10].

(3) $$\begin{array}{c} E_{\alpha }{}^{⟶}={\left|Cf^{⟶}.P_{\alpha }{}^{⟶}-P^{⟶}\right|}^{⟶},\\ E_{\beta }{}^{⟶}={\left|Cf^{⟶}.P_{\beta }{}^{⟶}-P^{⟶}\right|}^{⟶},\\ E_{\delta }{}^{⟶}={\left|{\text{Coef}}^{⟶}.V_{\delta }{}^{⟶}-V^{⟶}\right|}^{⟶},\\ P_{1}{}^{⟶}=P_{\alpha }{}^{⟶}-a_{1}{}^{⟶}.E_{\alpha }{}^{⟶},\\ P_{2}{}^{⟶}=P_{\beta }{}^{⟶}-a_{2}{}^{⟶}.E_{\beta }{}^{⟶},\\ P_{3}{}^{⟶}=P_{\delta }{}^{⟶}-a_{3}{}^{⟶}.E_{\delta }{}^{⟶},\\ P{\left(t+1\right)}^{⟶}. \end{array}$$

The optimal control process is carried out by the grey wolf's varied positioning as it approaches the prey.

#### 2.1.3. SRA (Sequential Randomized Algorithm)

SRA (sequential randomized algorithm) is an optimization algorithm that requires less time to process. SRA employs a suboptimal strategy of subdividing the problem into numerous subdivisions. By reducing the parameter values, constrained outputs are avoided; this ultimately results in a feasible solution. The SRA procedure entails the following steps: (1) initialization, (2) revision, (3) design, and (4) validation.

Iteration is kept to zero during the initialization process by using desiring as the likelihood. The process's total simulation count is set to *δ*. Following each iteration, the update processes should occur in order to fine-tune the optimal value given by Iter=Iter+1, *N*_iter_=*N*iter/Iter_*t*_, where *N* is the explicit sample bound chosen for the optimization procedure. The term *Q* refers to the uncertainty associated with a sample set, that is, *q*_*d*_={*q*_*d*_^(1)^ … *q*_*d*_^(*N*_iter_)^} the process by which the design of *N*_iter_ is calculated [11]. This procedure is manipulated by testing the random convex problem contained within it. Once it is determined that the optimization is not feasible, the iteration is updated and advanced to the next for further tuning. Each iteration is validated for the purpose of determining the feasibility by

(4) $$\begin{array}{c} M_{k}>\left\lceil \frac{\propto \;\ln\ln\left(\text{iter}\right)+\text{Ln}\left(S_{kt}\left(\propto \right)\right)+\ln\left(1/\delta \right)}{\ln\ln\left(1/\text{One}-\varepsilon \right)}\right\rceil , \end{array}$$

where

(5) $$\begin{array}{c} S_{kt}\left(\propto \right)=\sum_{\text{iter}=1}^{{\text{iter}}_{t}}\frac{1}{{\text{iter}}^{\alpha }} \end{array}$$

is shown to be a hyperharmonic series that analyzes the viability variables.

#### 2.1.4. The BSOA (Brainstorming Optimization Algorithm)

The BSOA (brainstorming optimization algorithm) [12] is a population-based evolutionary algorithm that is used to rapidly arrive at the optimal solution. The L-curve phenomenon is used to drive this algorithm. Rather than tackling a single complaint with a single brain, this process employs multiple minds to optimize the solutions. The brainstorming session will aid the algorithm in determining the correct answers in a shorter amount of time.

The brainstorming procedure is as follows:

1. The algorithm randomly selects individuals to propose potential solutions during the optimization process. The number of possible solutions is proportional to the number of participants.
2. Individuals are chosen and grouped together through selection and decision-making.
3. Each member of the cluster is analyzed in order to determine the optimal solution.
4. Following that, these solutions were ranked according to their viability.
5. The predefined probability is applied to the clusters, and the likelihoods are compared to determine which has the lowest probability.
6. Once again, this collection of individuals is for the purpose of cluster formation.
7. Once more, the new generation is developed until the ideal position is reached.
8. As a result, there are some optimization techniques that are appropriate for the process.

## 3. Sample Selection Optimization of the Spider Monkey

SMO is an optimization technique inspired by spider monkey foraging behavior. When spider monkeys run out of energy, they start developing under the leadership of a global leader. When necessary, the leader in the world divides the organization into numerous local organizations; each headed by a state legislator. Following one round of experimentation, the group shares its findings, or the leader relocates to an area with abundant food assets (i.e., optimal result) [13]. Thus, the global leader advances the performance analysis based on the aggregated results of the exploratory phase, whereas local leaders advance based on their community. Exploration in small groups improves foraging efficiency, and the presence of other monkeys mitigates location bias. As a result, SMO locates the optimal configuration rapidly and easily even when avoiding local maxima. SMO is advantageous for locating a specific point within the same constant environment. In WSNs, on the other hand, nodes have distinct locations. As a result, discovery is halted if no base station is found in a particular location during each round. Similarly, if the nearest point is chosen as the location to explore, inclusion is necessary to determine the nearer node. Rather than concentrating on specific locations, the SMO recommended using random samples to determine the most practicable samples. If the sampled population is made up of nodes, the outcomes are node locations. As a direct consequence, the issue of experimentation failing due to the unavailability of nodes in the optimizing location is settled [14].

## 4. Probability of Sampling

A sampling probability is used to select samples from the population. This probability is significant because, despite the lack of precise information about the effective product, the correlating distribution allows us to infer the expected samples. If the sampling probability is determined using the weight in SMO, the expected value may become zero or even negative. As a result, the recommended sampling-based SMO's weight must be informed consent to serve as the probability of sample selection [15]. The sampling probability is required to maintain samples for three phases of discovery: community official, world leader, and local leader decision. The local leader's phase is defined as follows:

(6) $$\begin{array}{c} {\text{SM}}_{i}^{\text{new}}\leftarrow \text{Rand}\left(0,1\right)\times \left(\text{LL}-{\text{SM}}_{i}\right)+\text{Rand}\left(-1,1\right)\times \left({\text{SM}}_{r}-{\text{SM}}_{i}\right),\;i=\left(1,2,\ldots ,N\right), \end{array}$$

where *N* denotes this same amount of spider monkeys, SM_*i*_^new^ denotes the spider monkey's location *i*^th^, and *i* denotes this same *i*^th^ spider monkey's highly satisfying. SM_*i*_ indicates the current location Rand(0,1) of the spider monkey, a random value between 0 and 1; LL indicates the location of a local official; and SM_*r*_ indicates the location of a randomly selected spider monkey from the same group. Equation (6) is stated in the following manner for the purpose of weights:

(7) $$\begin{array}{c} {\text{SM}}_{i}^{\text{new}}\leftarrow \left(1-\text{Rand}\left(0,1\right)-\text{Rand}\left(-1,1\right)\right)\times {\text{SM}}_{i}\\ +\text{Rand}\left(0,1\right)\times \text{LL}+\text{Rand}\left(-1,1\right)\times {\text{SM}}_{r},\;i=\left(1,2,\ldots N\right). \end{array}$$

The weights of SM_*i*_, LL, and SM_*r*_ are calculated as follows:

(8) $$\begin{array}{c} w_{{\text{SM}}_{i}}=1-\text{Rand}\left(0,1\right)-\text{Rand}\left(-1,1\right),\\ w_{\text{LL}}=\text{Rand}\left(0,1\right);w_{{\text{SM}}_{r}}=\text{Rand}\left(-1,1\right). \end{array}$$

Both *w*_SM_*i*__ and *w*_SM_*r*__ could indeed take low traits, and *w*_SM_*r*__ has the potential to be completely eliminated, as its average value is 0. To avoid these issues, we randomized the weights using the logistic softmax function. This function has been used in a number of recent studies to make meaningful selections, including Boltzmann exploration, neural network-based classification, reinforcement learning, and statistical modelling testing [16]. The logistic softmax is composed of complex numbers, which effectively eliminates the possibility of negative or zero weights:

(9) $$\begin{array}{c} \text{softmax }\left(w_{1},w_{2},w_{3},w_{4},\ldots w_{j},\ldots w_{m}\right)=\left(\frac{\text{exp}\left(w_{1}\right)}{\sum_{j=1}^{M}\text{exp}\left(w_{j}\right)},\frac{\text{exp}\left(w_{2}\right)}{\sum_{j=1}^{M}\text{exp}\left(w_{j}\right)},\ldots \frac{\text{exp}\left(w_{j}\right)}{\sum_{j=1}^{M}\text{exp}\left(w_{j}\right)},\ldots \frac{\text{exp}\left(w_{M}\right)}{\sum_{j=1}^{M}\text{exp}\left(w_{j}\right)}\right), \end{array}$$

where *M* denotes the total weights and *w*_*j*_ denotes the weight *j*. As a result, we will refer to the sampling likelihood as the softmax weight training in equation (7):

(10) $$\begin{array}{c} {\text{Prob}}_{\text{LLP}}\left(P_{1},P_{2},P_{3}\right)=\text{softmax }\left(w_{{\text{SM}}_{i}},w_{\text{LL}},w_{{\text{SM}}_{r}}\right). \end{array}$$

The assumption of weight training (*E*[Weight]), the sampling probability notation, and the sampling probability expectation (*E*[Probability]) for each development stage are listed in Table 1. Similarly, to the approach, distinct spider monkeys are chosen for each phase. In the strength column of Table 1, the lines are used: SM_*i*_ denotes the *i*^th^ spider monkey and LL denotes the local leader. The global leader is denoted by GL, and a randomly selected monkey from the same collective is denoted by SM_*r*_. This monkey serves as the village chief during the village chief phase and as the world leader during the global phase [17].

We intend to select CHs that are typically multiple. As a result, the sample size NS is greater than 1, so each spider monkey has a likelihood for each of these NS elements. As a result, instead of a single variable, each weight (Table 1) must be expressed as an array:

(11) $$\begin{array}{c} W_{{\text{SM}}_{i}}^{\text{NS}}=w_{{\text{SM}}_{i}}\times V_{\text{NS}}, \end{array}$$

where *V*_NS_=[1111 … 1] ∈ *R*^NS^, where *W*_SM_*i*__^NS^ is a weight array that includes NS occurrences of *W*_SM_*i*__, where *W*_SM_*i*__ is a weight array that contains NS occurrences of *W*_SM_. The following equation is modified:

(12) $$\begin{array}{c} {\text{Prob}}_{\text{LLP}}\left(P_{1},P_{2},P_{3},P_{4},.,.,.,P_{j}.,.,.,P_{M}\right)=\text{softmax}\left(w_{{\text{SM}}_{i}}^{\text{NS}},w_{\text{LL}}^{\text{NS}},w_{{\text{SM}}_{r}}^{\text{NS}}\right), \end{array}$$

where *M* denotes the selection of sampling likelihoods and because each spider monkey contains NS elements, *M*=NS × 3.

## 5. Algorithm for Optimization

As concerning traditional SMO, the approach is divided into seven phases: postprocessing, community politician, world leader, nearby trying to learn, global leader trying to learn, neighbouring decision, and worldwide decision. In comparison with conventional sampling SMO, random sampling SMO updates the discovery samples and the discovery location on a continual basis [18].

**Figure 1:**
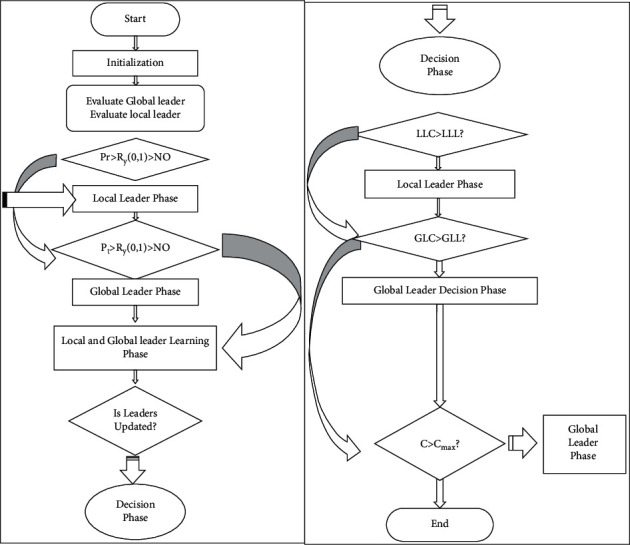
SMO based on sampling process.

A sample is denoted in the following manner:

(13) $$\begin{array}{c} \text{Sample}\left(\text{POP}=\left[S_{j}\right],NS,\;\text{Prob}=\left(P_{j}\right)\right),\;j=\left(1,2,3,\ldots ,M\right), \end{array}$$

where Sample denotes the sample, POP denotes the inhabitants (sampling candidate group), NS denotes the number of samples (in this case, the number of CHs), and Prob denotes the probability sampling array. Due to the fact that each element in set POP has its own possibility for sample selection, both sets are *M* in length, with each element being indexed by *j*.  Figure 1 illustrates a sampling-based SMO. As illustrated, the sampling-based SMO consists of seven phases: data preprocessing, local leader phase, global leader phase, local leader learning phase, global leader learning phase, local leader learning phase, and multiple decision phases. The subsets go into greater detail about each of these three phases [19].

### 5.1. Preprocessing

Initialization is the first step in exploration. In this phase, sampling is replicated *N* (swarm size) times to determine the required sample size for each spider monkey:

(14) $$\begin{array}{c} {\text{SM}}_{i}=\text{Sample}\left(U,NS,U\left(0,1\right)\right),\;i=\left(1,2,3,4,\ldots N\right), \end{array}$$

where *U* denotes the discovery universe (i.e., a set usually contains all aspects that can be sampled); *U*(0,1) denotes the uniform dispersion between 0 and 1, indicating that all elements have the same benefits in the form; and SM_*i*_ denotes the *i*^−th^ spider monkey's samples [20]. A spider monkey examines the cost function of samples. Then, the individuals with the highest fitness functions are chosen to serve as the initial global and local leaders.

### 5.2. Phase of the Local Leader

**Figure 2:**
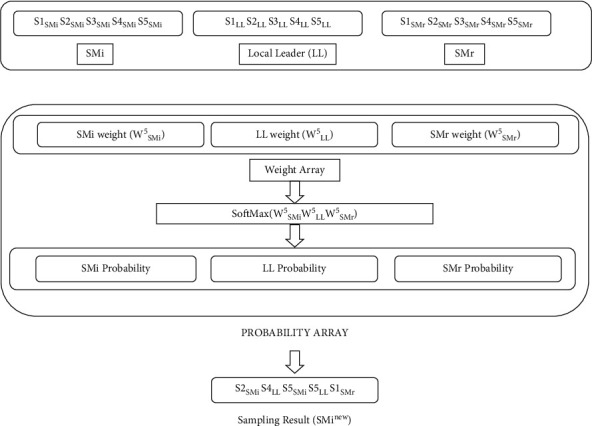
Phase of the local leader in the sampling-based SMO.

Each spider monkey SM_*i*_ releases its local leader LL and randomly chosen spider monkey SM_*r*_ samples, all of which are members of the very same group, throughout that phase:

(15) $$\begin{array}{c} {\text{SM}}_{i}^{\text{new}}\leftarrow \left\{\text{Samples}\left(\left\{X|X={\text{SM}}_{i}\cup \text{LL}\cup {\text{SM}}_{r}\right\},NS,\text{softmax}\left(W_{{\text{SM}}_{i}}^{NS},W_{LL}^{NS},W_{{\text{SM}}_{r}}^{NS}\right)\right),\;\text{if pr}>\text{Rand}\left(0,1\right){\text{SM}}_{i},\text{otherwise}\right., \end{array}$$

where {*X|X*=SM_*i*_ ∪ LL ∪ SM_*r*_} denotes the population; the softmax denotes the sampling probability, which would be given by equation (12); and pr denotes the perturbation rate, which generally increases from 0.1 to 0.4 as the number of iterations increases. This can be compared to the sampling illustration and described as follows [21]:

(16) $$\begin{array}{c} {\text{pr}}_{C+1}={\text{pr}}_{C}+\frac{0.4-0.1}{C_{\text{max}}},\;{\text{pr}}_{1}=0.1, \end{array}$$

where *C* is the current iteration number and *C*_max_ is the total number of iterations. The procedure for the local leader phase is depicted in Figure 2, where NS=5 and *S*1 − *S*5 reflect samples from each spider monkey (SM_*i*_, LL, and SM_*r*_) [22]. This value is sampled 5 times in almost every spider monkey *NS*, resulting in a total of 15 elements. The sample population contains 15 components due to the fact that 5*NS* samples were taken from the local leader phase. Because samples are collected during the local leader phase, the percentage of spider monkeys per spider monkey is also 5NS determined throughout that phase.

### 5.3. Phase of the Global Leader

Thus, every spider monkey needs to update its samples during this phase, using samples from the global leader GL and a selected randomly monkey SM_*r*_ as follows:

(17) $$\begin{array}{c} {\text{SM}}_{i}^{\text{new}}\leftarrow \left\{\text{Samples}\left(\left\{X|X={\text{SM}}_{i}\cup \text{GL}\cup {\text{SM}}_{r}\right\},\text{NS},\text{softmax}\left(W_{{\text{SM}}_{i}}^{\text{NS}},W_{\text{GL}}^{\text{NS}},W_{{\text{SM}}_{r}}^{\text{NS}}\right)\right),\;\text{if pr}>\text{Rand}\left(0,1\right){\text{SM}}_{i},\text{otherwise}\right.. \end{array}$$

As illustrated in equation 17, each spider monkey uses probability *P*_*i*_ to determine whether to update its samples. A higher fitness value indicates attained to the global leader, and the likelihood varies according to the number of iterations:

(18) $$\begin{array}{c} P_{i}=0.9\times \frac{{\text{Fitness}}_{i}}{\text{MAX}\left(\text{Fitness}\right)}+0.1, \end{array}$$

where Fitness_*i*_ is the fitness value of the *i*^th^ spider monkey and MAX(Fitness) is the maximum value of the overall fitness value.

During this phase, each local leader must update its samples with the finest samples obtained from the exploration results of the local group members. If a local leader's sample size remains constant, the local leader count, LLC, is increased by 1. The global leader updates its samples during this phase, using the best samples from all of the members' exploration results. If the sample size of a global leader remains constant, the global leader count, GLC, is increased by 1. When the LLC exceeds the local leader limit, LLL, local leaders alter the composition of the local group's membership samples [23]. Additionally, each member considers both global and local leader samples concurrently in order to create new samples for the purpose of exploring or initializing samples, as described in equation (13):

(19) $$\begin{array}{c} {\text{SM}}_{i}^{\text{new}}\leftarrow \left\{\text{Samples}\left(\left\{X|X={\text{SM}}_{i}\cup \text{GL}\cup \text{LL}\right\},\text{NS},\text{softmax}\left(W_{{\text{SM}}_{i}}^{\text{NS}},W_{\text{GL}}^{\text{NS}},W_{\text{LL}}^{\text{NS}}\right)\right),\text{ if pr}>\text{Rand}\left(0,1\right)\text{Initialization},\text{otherwise}\right., \end{array}$$

where pr is equal to the value defined in equation (16). This phase enables members of the local group to examine additional samples.

When the GLC exceeds the global leader limit, GLL, the global leader separates the group into several local groups, each with its own local leader. The number of local groups, LG, is enhanced by 1 in a sampling-based SMO until it reaches its maximum, MG.

### 5.4. Protocol for Choosing a CH

Through CH selection, the proposed method aims to increase the energy efficiency of WSNs. As a result, we considered a variety of factors that could affect energy efficiency. Then, we compared the performance of various protocols together under the conditions [24].

### 5.5. Model of a Network

Within the WSN, sensor networks have always been generated randomly within a square area. To determine energy efficiency, the following proposed protocol was used:

1. Each sensor node is assigned a unique identifier (ID).
2. The area includes one BS that is not contained within the WSN's square perimeter.
3. All cluster heads and the BS continue rising in their preexisting positions.
4. During the interaction's initialization phase, the BS and sensors declare their locations. As a result, the BS is informed of adjacent sensor nodes (covered nodes).
5. Due to the sensor nodes' particular features, they might still share or charge energy.
6. When the sensor nodes' available energy is depleted (Figure 4), they are never used once more.
7. There is no consideration of considerations that obstruct the transmission of data or damage WSN nodes.

### 5.6. Energy Calculator

Energy is consumed in three processes in the WSN: reception for the data transmission, and the consumed energy is denoted by the acronyms *E*_*TX*_ for data transmission, *E*_*RX*_ for reception, and *E*_*DA*_ for aggregation [25]. Unlike *E*_*DA*_ that remains constant over time, the values of *E*_*TX*_ and *E*_*RX*_ are situation-dependent. *E*_*TX*_ is dependent on the distance *d* between the sensor nodes and transmitter, and different meanings are used; obviously, it depends on whether *d* is greater than or less than the threshold distance d0. When *d* < d0, the free-space prototype is used; otherwise, a mole of the multipath is used:

(20) $$\begin{array}{c} E_{TX}=\left\{l\times E_{\text{elec}}+l\times \varepsilon_{fs}\times d^{2}\text{ if }d<\text{d}0l\times E_{\text{elec}}+l\times \varepsilon_{\text{mp}}\times d^{4}\text{otherwise}\right., \end{array}$$

where *E*_elec_ is the electrical power ability to change one bit of data to a signal, *l* is the data size, and *ε*_fs_ and *ε*_mp_ are the power consumptions that are used by the free-space and multichannel models, respectively, and which acts as methods for evaluating *d*0 [26]:

(21) $$\begin{array}{c} d0=\sqrt{\left(\frac{\varepsilon_{\text{fs}}}{\varepsilon_{\text{mp}}}\right)}, \end{array}$$

where *E*_*RX*_ is the energy ability to change a received data to data and *E*_elec_ is also absorbed during the same process; similarly to *E*_*TX*_, *E*_*RX*_ is proportional to the length of the collected data: *E*_*RX*_=*l* × *E*_elec_.

### 5.7. Objective

To maximize the energy productivity of WSNs, it is critical to select the appropriate CHs in organizational structure clustering protocols. When selecting CHs, the first critical factor to consider is their distribution, as CHs clustering sensors on one side increases the distance between sensor nodes and the. As a result, the gain from transmission distance to the closest centroid is reduced for each node CHs. To keep transmission power usage at the CH nodes to a low, it must be spread correctly. The appropriate comparison of arithmetical CH nodes is necessary at this point. An adequate distribution of CHs should result in both comparable coverage regions across CHs and availability for each devices.

(22) $$\begin{array}{c} A_{\text{Cover}}=\frac{d_{\text{far}}^{2}\times \pi }{NS}, \end{array}$$

where *A*_Cover_ is the coverage area and *d*_far_^2^ is the squared width between the node's farthest node and its midpoint. Thus, all nodes are contained within *d*_far_^2^, the circular area delineated by the nodes' midpoints, and any node within the circle delineated by the CH has a range smaller than the radial distance of that circle CH [28]. Furthermore, trying to compare distances is simpler than assessing whether a node is contained within a circle:

(23) $$\begin{array}{c} R_{\text{Cover}}=\sqrt{\frac{A_{\text{Cover}}}{\pi }},\\ R_{\text{Cover}}=\sqrt{\frac{d_{\text{far}}^{2}\times \pi }{\text{NS}\times \pi }},\\ R_{\text{Cover}}=\frac{d_{\text{far}}^{2}\times \pi }{\sqrt{\text{NS}}}, \end{array}$$

where *R*_Cover_ is the distance (radius) used to evaluate if a node is within *A*_Cover_ coverage area. The set of nodes encompassed by the *k*^th^CH is denoted by

(24) $$\begin{array}{c} {\text{Cover}}_{k}={\text{Node}}_{\text{ID}}|\text{Distance}\left({\text{Node}}_{\text{ID}},CH_{k}\right)<R_{\text{Cover}},\;k=\left(1,2,3,\ldots ,\text{NS}\right)\forall \text{ ID}, \end{array}$$

where Distance(Node_ID_, CH_*k*_) denotes the distance between the node identified uniquely by CH and the *k*^th^CH. Take note that *R*_Cover_ perceives the entire coverage area to be evenly split. Thus, the greater the number of nodes covered by the CH, the better the distribution [29]. As a result, an objective function can be expressed as follows:

(25) $$\begin{array}{c} F_{1}=\left|\cup_{k=1}^{NS}{\text{Cover}}_{k}\right|, \end{array}$$

where | | denotes the set's cardinality (i.e., the number of elements) and the union precludes counting nodes covered by multiple CHs.

Another factor to consider is node energy that is frequently used to determine the CH to use. The node energy is separated into transmission, reception, residual, and cluster formation energy, which are all combined back into the reserve energy (i.e., the energy consumed and left when a node becomes a CH). If a node with a very low reserve energy capacity is chosen as CH, it can be decimated without receiving all data from neighbouring nodes, dramatically reducing stability [30]. As a result, CHs are preferred to have a large reserve of energy. The reserve energy's objective function is denoted by the symbol.

(26) $$\begin{array}{c} F_{2}=\sum_{k=1}^{\text{NS}}{\text{RES}}_{k}-\left(|{\text{Cover}}_{k}\times E_{RX}+E_{DA}+E_{TX}\right), \end{array}$$

where RES_*k*_ denotes the energy consumption of the *k*^th^CH and *E*_*TX*_, *E*_*RX*_, and *E*_*DA*_ denote the energy required for data transmitting, reception, and aggregation, respectively, as discussed in Section 4. The optimization problem, *F*_obj_, considers both *F*_1_ and *F*_2_ simultaneously via their weighted sum in order to balance their contributions during optimization [31]. We used min-max normalization to establish the objective function. Because the number of covered nodes and deposit energy will always be greater than 0, the lower of both the two best fitness is 0. As a result, the objective function that normalizes the two objective values is as follows:

(27) $$\begin{array}{c} F_{\text{obj}}=w_{F_{1}}\times \frac{F_{1}}{\text{MAX}\left(F_{1}\right)}+w_{F_{2}}\times \frac{F_{2}}{\text{MAX}\left(F_{2}\right)}, \end{array}$$

where *w*_*F*_1__ and *w*_*F*_2__ are the respective fitness values' weights (both set to 0.5 in this study) [32]. As higher *F*_1_ and *F*_2_, CH selection model will be better with Maximize(*F*_obj_).

### 5.8. Protocol for CH Selection

**Figure 3:**
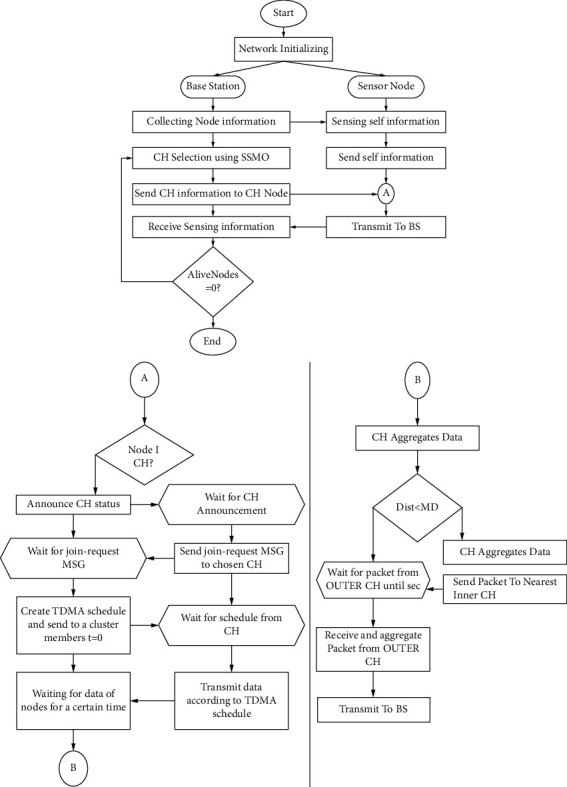
Flowchart of spider monkey optimization using sampling and energy-smart clustering the smart spider monkey optimization (SSMO) protocol: (a) communication between the base station (BS) and nodes, (b) communication between cluster heads (CHs) and nodes (SSMO: sampling-based SMO and TDMA: time division multiple access).

We describe the proposed ESMO procedure for CH selection in specifics in this subchapter. The data transmission between the BS and the nodes are depicted in Figure 3. When a WSN is formed, each sensor node transmits initial data to the base station (BS), which includes the node's ID and location. Using the received data, the BS selects CHs via sampling-based SMO [33]. The BS notifies the CHs of their selection as CHs and distributes synchronization data. This process is depicted in Figure 3(a) as a line connecting the grey circle A. Following CH selection, data transfer proceeds in a manner similar to that of LEACH − *C* and SMOTECP. As illustrated in Figure 3(b), the CHs notify their covered nodes of the selection and await confirmation via an acknowledgment (ACK) signal. The CHs that receive the ACK signal transmit a schedule for time division multiplexing to the covered nodes and gather data for a specified period of time before transmitting the data to the next CH or BS [34]. The CHs that communicate with other CHs are referred to as outer CHs, while the CHs that communicate directly with the BS are referred to as inner CHs. This distinction is made by calculating the median of the ranges (MD) between the CH*s* as well as the BS depicted in Figure 3 as the grey circle B's flowchart. The data collection using WSN at the BS is replicated until all nodes' energy is depleted [35].

### 5.9. Discussion from the Results

**Figure 4:**
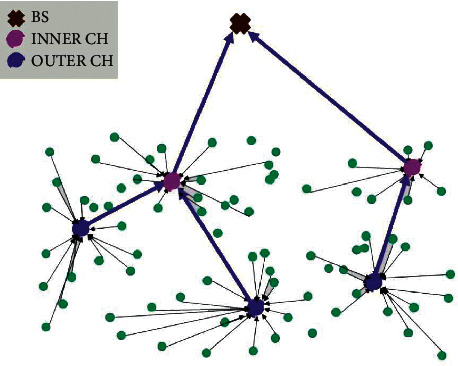
The topology of the SSMO network and the distribution of CH.

Through integration on Python 3.6 using applicable library functions such as Network, Numpy, and Matplotlib, we compared the proposed SSMO (as shown in Figure 4) to centralized protocols such as LEACH-C, PSO-C, and SMOTECP. The experiment was conducted in both a relatively homogeneous setup, where all nodes had the same initial energy, and a heterogeneous setup, where nodes had varying initial energies [36]. The experimental results are presented in terms of network topology, lifetime, energy is consumed, and energy efficiency.

### 5.10. Setup for the Experiment

We were able to compare the efficacy of the reviewed protocols fairly by using similar experimental settings to those used in the majority of studies. The experimental parameters are summarized in Table 2. Except for the BS location, which varied according to the purpose of each analysis, the periods in question were compatible with those reported in the literature. For example, it was set to (50, 50) *m*, (50, 150) *m*, and (50, 175) *m*. The distance between both the node and the BS varied with its location, which provided information about the transmission model (equation (20)). Calculating d0 using equation (21) and the *fs* and *mp* values from Table 2 resulted in an 87.706 m threshold. When the BS was located at (50, 50) m, the distance between it and the furthermost node was 70.71 m. As a result, the free-space model was used to communicate between all nodes. When the BS was located at (50, 175) m, the distance between each node was between 50 and 182 m, indicating that the multipath model was used by the majority of nodes. We located the BS at (50, 150) m in order to achieve a 1:2 ratio between the available and multimode models, which enables us to evaluate both. The SMOTECP protocol specified the location of the BS as (50, 50) m. Thus, when the BS was located at (50, 150) m, all CHs were classified as external CHs by SMOTECP. As a result, the selection process for inner and outer CHs should be altered. After selecting the CHs as explained in, the inner and outer CHs were determined as illustrated in Figure 3(b). Likewise, SSMO requires the swarm parameters listed in Table 3 for sampling-based SMO. Furthermore, these parameters are used for PSO-C and SMOTECP.

### 5.11. Evaluation of Performance

The initial topology of the network following the creation of nodes illustrates the topology for the evaluated protocols following CH selection. The network topology analysis's output shows the transmission path and distance. Due to the fact that the transmission distance is roughly equal to the transmission energy, the energy demand can be estimated using the entire network result. In comparison to other protocols based on swarm intelligence, LEACH-C returned an inappropriate CHs distribution. Indeed, LEACH-C enabled transfer between certain nodes and CHs over long distances. The other protocols recovered a similar amount of energy from the nodes. SSMO consumed the same quantity of electricity as the other procedures but did so in a more even distribution in Figure 4. As a result, SSMO could more effectively distribute the network's energy consumption.

The simulated results for the heterogeneous setup, where all nodes in the WSN started with a 1 J initial energy. SSMO had a later first node death than the other protocols, resulting in a more stable network for a longer period of time. SSMO also increases the network's lifetime, as the network's half and final devices survive the longest. The SSMO network's final node to die occurred last, indicating an enhanced network lifetime. Again, SSMO outperformed the other protocols in terms of energy consumption. In the homogeneous setup, Table 4 shows the execution rounds during which the first, half, and final nodes died. After determining the expected CH position using PSO-C, the nearest node was calculated, which appeared to significantly reduce energy efficiency in the heterogeneous configuration compared to the homogeneous configuration. In the heterogeneous configuration, this SSMO requires less power than the other protocols [37]. In the heterogeneous setup, Table 5 shows the execution rounds in which the first, half, and final nodes died.

The stable duration, unstable period, and lifetime of the network are listed in Table 6, where stability is described as the survival of all sensor nodes. When compared to LEACH-C, PSO-C, and SMOTECP, SSMO increased the transmission delay by 20%, 12.9%, and 7.4%, respectively. Similarly, network lifetimes were increased by 12.3%, 5.6%, and 3.5%, respectively. When contrasted to LEACH-C, PSO-C, and SMOTECP, SSMO increased the battery lifetime by 60%, 41.6%, and 2.2%, respectively, under heterogeneous conditions. Additionally, the network's lifetime was increased by 2.6%, 2.2%, and 0.7%, respectively. By and large, the results indicate that SSMO significantly improved network stability and lifetime when particularly in comparison to other CH selection processes.

## 6. Conclusion

The smart operation of clustered WSN protocols depends on CH selection. Several previous research studies determined the best CH placement by choosing the nearest nodes as CHs. The difference in position between the desired and real CH node location, we reasoned, may risk energy efficiency. As a result, we developed SSMO, a method for choosing CHs that uses sampling to account for real node positions. The best CHs are found by sampling and configuring them with a specific SMO algorithm that eliminates divergence between the desired and actual CH node positions, resulting in increased energy efficiency. The experiment employs two distinct experimental designs to assess the proposed method: homogeneous and heterogeneous. When compared to other similar protocols in a homogeneous configuration, SSMO increased the lifetime of the network and stability by an average of 12.22% and 6.9%, respectively (LEACH-C, PSO-C, and SMOTECP). Similarly, in the heterogeneous setup, SSMO increased the network's lifetime and stability by an average of 32.65% and 1.8%, respectively. The suggested SSMO's great performance was shown experimentally by extending the network's lifetime and stability by intelligently utilizing energy. As a consequence, current conflicts may be resolved by using SSMO to convert from location-based to node-based CH selection while also boosting network performance.

## Figures and Tables

**Table 1:** Probability definitions for sampling based on the sample update phase.

Phase	Strength	*E* (weight)	Notation
Phase of the local leader	wSMi	0.52	Softmax (wSMi, wLL, wSMr)
	wLL	0.52	
	wSMr	0	
Phase of the global leader	wSMi	0.52	Softmax (wSMi, wGL, wSMr)
	wGL	0.52	
	wSMr	0	
Phase of local-leader decision	wSMi	0	Softmax (wSMi, wGL, wLL)
	wLL	0.52	
	wGL	0.52	

**Table 2:** Criteria of the network for evaluating CH selection protocols.

Parameter	Value
Node count	100
Size of the network	100 × 100 m
Base statio's location	(50, 150) m
Initial energy that is not homogeneous (**E**_**hete**_)	(0.5, 1) J
Initial energy that is homogeneous (**E**_**0**_)	1 J
Radio-frequency electronic energy (**E**_**elec**_)	50 nJ/bit
Parameter for the free-space channel (*ε*_**fs**_)	10 pJ/bit/m^2^
Parameter for multipath channels (*ε*_**mp**_)	0.0013 pJ/bit/m^4^
Efforts devoted to data aggregation (**E**_**DA**_)	5 nJ/bit
Probability of CH selection (**P**_**CH**_)	5%
The maximum length of a message sent from a node to a CH	2,800 bits
The length of packets transmitted from the CH to the BS	6,400 bits

**Table 3:** Optimization of swarm parameters.

Parameter	Value
Swarm dimensions	40
Iterations to a maximum (**Cmax**)	100
The maximum number of groups possible	4
Limitation on global leaders	10
Limitation on local leaders	20

**Table 4:** Nodes that are still alive after a protocol implementation round in a homogeneous setup.

Alive nodes (%)	LEACH-C	PSO-C	SMOTECP	SSMO
99 (FND)	2069	2200	2313	2484
90	2197	2312	2377	2795
80	2266	2410	2407	2925
70	2309	2449	2427	2976
60	2360	2512	2453	3019
50 (HND)	2395	2622	2472	3030
40	2449	2684	2495	3039
30	2512	2808	2521	3048
20	2609	2861	2597	3056
10	2684	2878	2697	3060
0 (LND)	2729	2903	2963	3065

**Table 5:** Nodes that are still alive after a protocol execution round in a heterogeneous setup.

Alive nodes (%)	LEACH-C	PSO-C	SMOTECP	SSMO
99 (FND)	1275	1441	1997	2040
90	1492	1566	2069	2139
80	1609	1650	2077	2167
70	1670	1715	2087	2185
60	1728	1914	2094	2201
50 (HND)	1815	2037	2100	2208
40	1895	2115	2103	2214
30	1946	2141	2107	2216
20	1996	2162	2110	2220
10	2047	2173	2118	2221
0 (LND)	2168	2177	2209	2225

**Table 6:** Period and lifetime of network stability (measured in execution rounds) vary according to protocol and configuration.

Homogeneous setup				
Period	LEACH-C	PSO-C	SMOTECP	SSMO
Stable period	2069	2202	2314	2485
Unstable period	661	703	650	581
Lifetime	2731	2904	2964	3067
Heterogeneous setup				
Period	LEACH-C	PSO-C	SMOTECP	SSMO
Stable period	1275	1442	1998	2041
Unstable period	894	736	213	185
Lifetime	2169	2178	2210	2226

## Data Availability

The data used to support the findings of this study are available from the corresponding author upon request.
